# Consensus-based technical recommendations for clinical translation of renal T1 and T2 mapping MRI

**DOI:** 10.1007/s10334-019-00797-5

**Published:** 2019-11-22

**Authors:** Ilona A. Dekkers, Anneloes de Boer, Kaniska Sharma, Eleanor F. Cox, Hildo J. Lamb, David L. Buckley, Octavia Bane, David M. Morris, Pottumarthi V. Prasad, Scott I. K. Semple, Keith A. Gillis, Paul Hockings, Charlotte Buchanan, Marcos Wolf, Christoffer Laustsen, Tim Leiner, Bryan Haddock, Johannes M. Hoogduin, Pim Pullens, Steven Sourbron, Susan Francis

**Affiliations:** 1 Department of Radiology, Leiden University Medical Center, Leiden, The Netherlands; 2 Department of Radiology, University Medical Center Utrecht, Utrecht University, Utrecht, The Netherlands; 3 Department of Biomedical Imaging Sciences, Leeds Institute of Cardiovascular and Metabolic Medicine, University of Leeds, Leeds, UK; 4 Sir Peter Mansfield Imaging Centre, School of Physics and Astronomy, University of Nottingham, Nottingham, UK; 5 Department of Radiology, Translational and Molecular Imaging Institute, Icahn School of Medicine at Mount Sinai, New York, NY USA; 6 Centre for Inflammation Research, Queen’s Medical Research Institute, University of Edinburgh, Edinburgh BioQuarter, Edinburgh, UK; 7 Department of Radiology, Center for Advanced Imaging, NorthShore University Health System, Evanston, IL USA; 8 Centre for Cardiovascular Research, Queen’s Medical Research Institute, University of Edinburgh, Edinburgh BioQuarter, Edinburgh, UK; 9 Institute of Cardiovascular and Medical Sciences, University of Glasgow, Glasgow, UK; 10 Antaros Medical, Mölndal, Sweden; 11 MedTech West, Chalmers University of Technology, Gothenburg, Sweden; 12 Center for Medical Physics and Biomedical Engineering, MR-Centre of Excellence, Medical University of Vienna, Vienna, Austria; 13 Department of Clinical Medicine, MR Research Centre, Aarhus University, Aarhus, Denmark; 14 Department of Clinical Physiology, Nuclear Medicine & PET, Rigshospitalet, Copenhagen University Hospital, Glostrup, Denmark; 15 Department of Radiology, University Hospital Ghent, Ghent, Belgium; 16 Ghent Institute of Functional and Metabolic Imaging, Ghent University, Ghent, Belgium

**Keywords:** Kidney, T1 relaxation, T2 relaxation, MRI, Biomarkers, Standardization, Consensus

## Abstract

To develop technical recommendations on the acquisition and post-processing of renal longitudinal (T1) and transverse (T2) relaxation time mapping. A multidisciplinary panel consisting of 18 experts in the field of renal T1 and T2 mapping participated in a consensus project, which was initiated by the European Cooperation in Science and Technology Action PARENCHIMA CA16103. Consensus recommendations were formulated using a two-step modified Delphi method. The first survey consisted of 56 items on T1 mapping, of which 4 reached the pre-defined consensus threshold of 75% or higher. The second survey was expanded to include both T1 and T2 mapping, and consisted of 54 items of which 32 reached consensus. Recommendations based were formulated on hardware, patient preparation, acquisition, analysis and reporting. Consensus-based technical recommendations for renal T1 and T2 mapping were formulated. However, there was considerable lack of consensus for renal T1 and particularly renal T2 mapping, to some extent surprising considering the long history of relaxometry in MRI, highlighting key knowledge gaps that require further work. This paper should be regarded as a first step in a long-term evidence-based iterative process towards ever increasing harmonization of scan protocols across sites, to ultimately facilitate clinical implementation.

**Electronic supplementary material:**

The online version of this article (10.1007/s10334-019-00797-5) contains supplementary material, which is available to authorized users.

## Introduction

There is an increasing need for the development of non-invasive imaging biomarkers to assess the influence of fibrosis and inflammation in the kidney. Renal disease often progresses unnoticed and clinical measurements such as estimated glomerular filtration rate (eGFR) and albuminuria tend to deteriorate late in the disease course. The application of MRI for non-invasive tissue characterization by voxel-wise mapping of longitudinal (T1) and transverse (T2) relaxation time of the kidney without contrast media, referred to as native T1 and T2 mapping, is a promising tool for predicting clinical outcomes in parenchymal renal disease and providing guidance in clinical decision-making. T1 and T2 relaxation times can be indicative of alterations in tissue composition such as fibrosis, oedema or cyst progression [1–3]. The ability of non-invasive tissue characterization could ultimately be used for better understanding of parenchymal renal disease and for the monitoring of novel drug effectiveness. However, one of the main challenges of research on new MRI biomarkers such as T1 and T2 mapping is the variability in measurement due to lack of standardization in patient preparation, hardware, data acquisition and post-processing.

The European Cooperation in Science and Technology (COST) Action Magnetic Resonance Imaging Biomarkers for Chronic Kidney Disease (PARENCHIMA, CA16103, https://renalmri.org) was established to share best practice and realize the full potential of renal MRI biomarkers. As part of the COST Action PARENCHIMA initiative a systematic review on T1 and T2 mapping was performed which indicated the lack of agreement in patient preparation, acquisition protocols and adequate patient selection, as well as widely accepted reference values [4]. Thus, there is a need for optimization and standardization of (multi-parametric) MRI protocols to increase the specificity of renal T1 and T2 mapping. In line with these aims, the COST Action PARENCHIMA has initiated a consensus project to define expert-based technical recommendations to harmonize imaging protocols and image analysis. This PARENCHIMA consensus project aimed to develop and apply a process for generating technical recommendations on renal MRI using Arterial Spin Labelling (ASL), Diffusion Weighted Imaging (DWI), Blood Oxygenation Level Dependent (BOLD), and T1 and T2 mapping, with a common seven-stage process was defined for attaining consensus across each, as outlined in a covering paper [5]. The technical recommendations outlined in this paper are intended to provide guidance on the current consensus of set-up of imaging protocols for researchers who are new to the field of renal T1 and T2 mapping or researchers who are interested in combining T1 and T2 mapping within a multi-parametric renal MRI protocol. However, these recommendations should not be interpreted as absolute, as specific research questions might require deviations from current proposed recommendations, and novel state-of-the art developments could bring new insights into scan acquisition protocols or image analysis. In addition, these recommendations focus on the application of T1 and T2 mapping for visualization, quantification and monitoring of parenchymal renal disease rather than for the characterization of focal renal lesions. Moreover, it is outside the scope of the current consensus project to define recommendations on phantoms and/or reference standards to use. But it must be highlighted that any systematic comparison of T1 and T2 mapping schemes should include phantom validation across a range of T1/T2 values. A number of commercially available phantoms with a number of test vials across reference in vivo T1 and T2 ranges are available (for example, the ISMRM/NIST phantom [6] or Eurospin test object TO5 (Diagnostic Sonar, Livingston, UK). These phantoms can be used to define reference T1 values from an inversion-recovery spin-echo series and T2 values using a Carr–Purcell–Meiboom–Gill (CPMG) sequence with which to assess the accuracy and precision of other T1 and T2 mapping schemes. Such phantoms have been used in harmonization studies, for example in the brain and heart, but to date, limited studies have reported inter-vendor or site measures associated with renal T1 and T2 mapping protocols. In this paper, we first provide a background overview of technical parameters related to renal T1 and T2 mapping at clinical field strengths (1.5 and 3 Tesla); in the methods section, we describe how the consensus project was performed; in the results section, the achieved consensus recommendations are discussed in detail; and in the discussion, we elaborate on the issues not achieving consensus, and identify areas for future research.

## Overview of technical parameters

### T1 mapping schemes

Three different approaches for T1 mapping have generally been implemented.

#### Classical inversion recovery (IR)

**Fig. 1:**
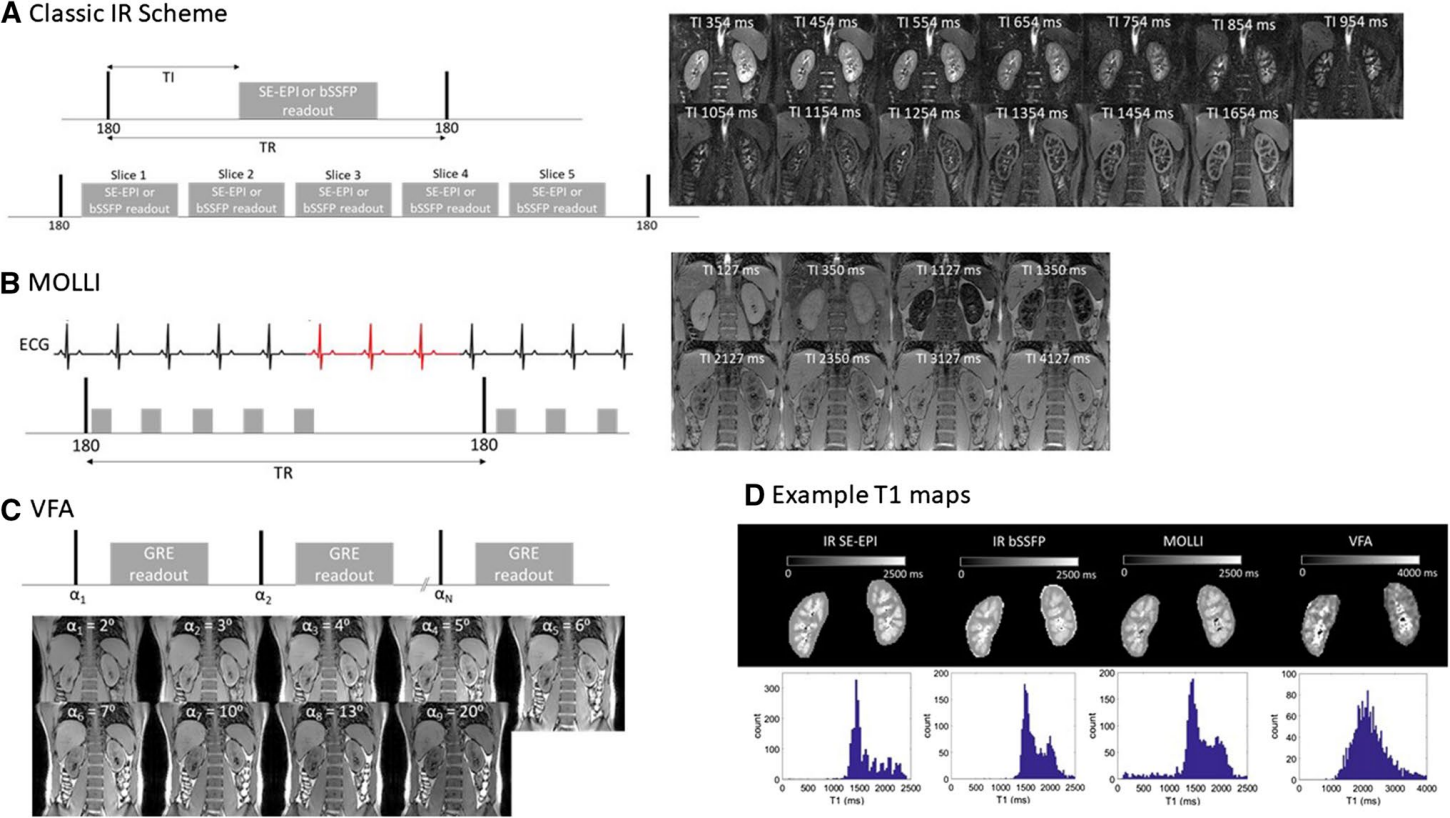
Renal T1 mapping acquisition schemes. (**a** Classic IR scheme, **b** MOLLI, **c** VFA) and example images and T1 maps. **a** Classic IR scheme illustrated here with a spin-echo EPI (SE-EPI) or balanced gradient echo/balanced steady state free precession readout. Traditionally, after an inversion pulse a single image readout is acquired after an inversion time (TI), this scheme is available across all vendors. In the *slice cycling* approach, the empty space is filled with readouts of different slices, as shown here. In the next TR, the slice ordering is shifted to acquire a different initial slice in a given TR. To increase the dynamic range of the TIs, a delay can be added between slice acquisitions. **b** MOLLI with a 5(3)3 scheme: after the first 180° inversion pulse 5 image readouts are acquired in 5 consecutive heart-beats followed by a 3 beat recovery period. After the second 180° inversion pulse, three image readouts are acquired. Note, for renal T1 mapping, rather than ECG gating (which is required for cardiac T1 mapping), a fixed spacing of 1 s between image readouts is recommended. **c** A spoiled gradient echo (GRE) image is collected at a number of flip angles in separate acquisitions from which a T1 map can be calculated

In this scheme, each repetition time (TR) contains a single 180° inversion pulse which, after a delay termed the inversion time (TI), is followed by a single readout. After waiting for full magnetization recovery, this is repeated for a number of TIs to accurately sample the IR curve. A single slice classic IR scheme can be implemented across all MR vendors. However, the disadvantage of this technique is that it is slow, with a single slice acquisition being dependent on the number of inversion recovery times used and the longest recovery time which is also field dependent. The total time can be reduced using partial post-readout recovery, but if data are collected respiratory triggered, then a full respiratory cycle must be allowed between inversion recovery times. A multislice version of the classic IR approach extends the scan time by a factor of the desired number of slices. Therefore, a number of alternative modifications have been proposed to accelerate this for multislice measurements. The simplest option is to follow the 180° inversion pulse by a multislice readout, as illustrated in Fig. 1a, but this can limit the dynamic range of the TI values across slices, especially for non-EPI-based readouts. An elegant solution to this problem is to use *slice cycling* [7, 8]. Here, instead of repeatedly sampling the same initial slice after the inversion pulse, one can iterate to sample a different slice. Thus, in the next TR, the order of slices is shifted (the first slice is now measured last) and so on until all slices have been measured at each timepoint. Consequently, the number of slices equals the number of timepoints. To extend the dynamic range of the TI, a delay can be inserted between the slice readouts. However, this approach is not generally available on commercial MR systems. A simplified version of *slice cycling* in which the slice ordering is changed from ascending to descending between TR periods can be a practical solution to implement on commercial systems [9].

#### Look-locker (LL) sequence and variants such as modified look-locker inversion recovery (MOLLI)

This is an attractive approach in which a given slice is repeatedly sampled after a single 180° inversion pulse [10, 11]. The original LL sequence consisted of repeated low flip-angle readouts of a given slice after the inversion pulse. In this way, a single-slice T1 measurement can be performed in a given TR, usually within one breath hold. An important consideration of this approach compared to classical inversion recovery is that the readouts influence the T1 recovery. Therefore, an ‘apparent T1’ is measured which has to be corrected to compute the ‘true T1’.

Variants on the Look-Locker sequence, like MOLLI, were developed for cardiac T1 mapping, where the image readout must be aligned with the cardiac phase [12]. Since MOLLI is widely available on commercial scanners within the cardiac package, it is now also being routinely used for renal T1 mapping [4]. In the original MOLLI implementation, a 3(3)3(3)5 scheme was proposed: 3 images acquired following an inversion pulse, a 3-heartbeat recovery period, an inversion pulse followed by a further 3 images and 3-heartbeat recovery period, and a third inversion pulse followed by 5 images [13]. However, there is some dependency of the measured T1 on the heart rate. More recently, a 5(3)3 scheme (Fig. 1b), which reduces the influence of heart-rate, because the recovery time following the first inversion is increased, has been implemented and is available on all MR vendors. A fixed spacing between acquisitions can be used instead of using cardiac triggering which is more appropriate for renal T1 mapping applications. This can be achieved on all commercial systems, either as an option or by turning on physiological simulation on the scanner when in research mode.

#### The variable flip angle (VFA) approach

This has been used for T1 mapping due to its ease of implementation on all commercial systems. This method does not use an inversion pulse, but instead collects spoiled gradient echo images at a number of different flip angles in separate acquisitions (Fig. 1c) from which a T1 map can be calculated [14]. However, in abdominal imaging and especially at higher field strength, the actual flip angle (B1 +) delivered to the abdomen will vary, altering the fitted T1. For the VFA scheme, a separate B1 map is thus required to correct for any B1+ inhomogeneity which can result in poor precision for the absolute assessment of native T1 [15], although acceptable when using the VFA scheme to measure a change in T1 to a challenge (such as inhalation of oxygen).

### T2 mapping schemes

**Fig. 2:**
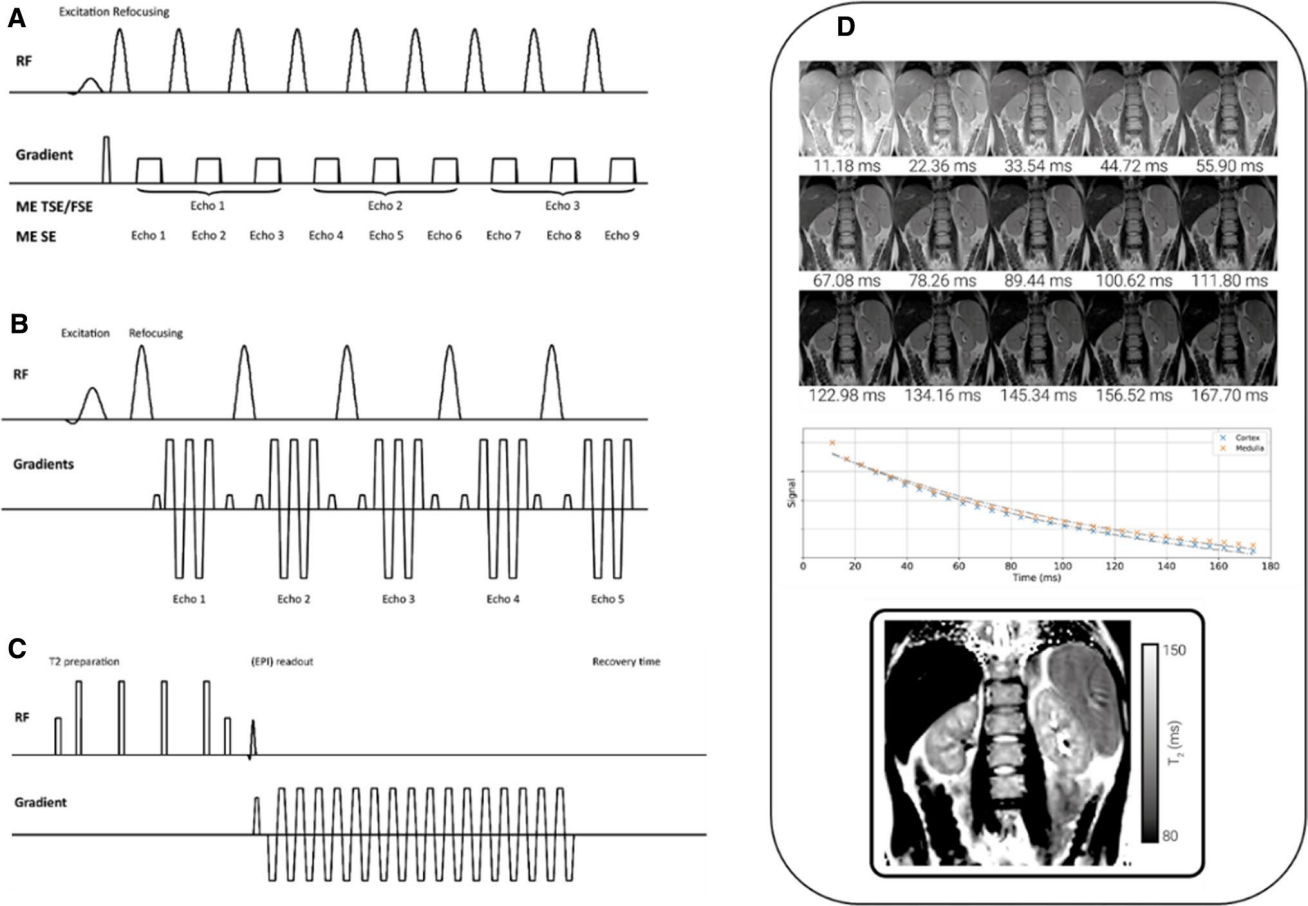
Acquisition schemes (**a** MESE, **b** GRASE, **c***T2 prep*) for renal T2 mapping, and example T2 maps using the GRASE scheme. **a** MESE: After a 90° excitation pulse, the transverse magnetization is repeatedly refocused by a train of 180° pulses, with a single k-line acquired after each refocusing pulse (here illustrated for 9 echoes). Multiple TRs are then needed to fill the entirety of k-space. For MESE, the number of refocusing pulses equals the number of images, each with a different T2 weighting, so every k-line acquired in a single TR is assigned to a different image. For TSE/FSE, a number of k-lines (here illustrated for 3 k-lines) are assigned to the same image, which consequently results in a slightly mixed T2 weighting. The more subsequent k-lines assigned to the same image (higher turbo-factor), the less defined is the T2 weighting of the resulting image. **b** GRASE: Contrary to MESE, multiple k-lines are acquired after every refocusing pulse using an EPI-like acquisition. **c***T2 prep*: A T2 preparation is immediately followed by a single-shot readout (in the image an EPI readout). Note that it is important to add some time after the readout to allow the longitudinal magnetization to recover before repeating the acquisition at the next effective echo time. **d** Example images for a GRASE T2 mapping scheme, with associated signal in the cortex and medulla and T2 map

For T2 mapping, the preparation consists at least of a combination of generally a 90° followed by 180° refocusing RF pulse. The most straightforward approach is a conventional *multi*-*echo spin echo (MESE)* sequence which acquires multiple T2 weightings of a given k-space line in turn (Fig. 2a). The MESE sequence can be accelerated using *turbo spin echo (TSE) or fast spin echo (FSE)* (Fig. 2a) In TSE/FSE, as the turbo-factor increases, the T2 weighting of the source image is slightly less defined, so high turbo-factors are not suitable for T2 mapping. Variants of the MESE scheme can be implemented on MR scanners of all vendors. Alternatively, a *gradient and spin*-*echo (GRASE)* sequence (Fig. 2b, d) can be used which contains both spin and gradient echo characteristics [16]. GRASE is much faster as compared to TSE/FSE and has a lower specific absorption rate (SAR); however, T2* effects are introduced, especially at higher acceleration factors.

MESE-based sequences have limitations in that they are sensitive to imperfect slice profiles, diffusion, flow and field inhomogeneities. To minimize such sensitivities, *T2 preparation modules (T2 prep)* can be used (as shown in Fig. 2c). Similar to the application of an inversion pulse prior to the readout in T1 mapping, here ‘*T2 prep’* modules are placed before a fast single-shot readout. Typical *T2 prep* modules include Carr–Purcell–Meiboom–Gill (CPMG) or four equally spaced composite refocusing pulses with Malcolm-Levitt phase cycling (MLEV4, Fig. 2c [17]), but this scheme is sensitive to B0 and B1 inhomogeneities [18–20]. Alternatively, a modified B1-insensitive rotation (mBIR-4) scheme [21] can be used, or schemes which use a pair (Silver–Hoult-pair) [22] or multiple adiabatic full passage (AFP) pulses [23]. The performance of these schemes have been compared for cardiac imaging [23, 24], but to our knowledge, a detailed comparison of different T2 mapping methods has yet to be performed for imaging the kidney. A sufficient recovery time between preparations must be allowed for full T1 recovery, else incomplete T1 recovery results in T1 weighting and errors in the T2 relaxation time measurements [25]. The main disadvantage of a ‘T2 prep’ scheme is the long acquisition time (TR x number of T2 weightings x number of slices). T2 mapping with T2 prep is not generally implemented across all commercial MR systems and may have a limited choice and number of different effective echo times.

### Readout strategy

In general, the image readout for a mapping scheme should be a stable 2D single-shot sequence with high SNR, which enables fast imaging, making the readout less sensitive to motion. In renal imaging, gradient or spin echo planar imaging (EPI), fast gradient-echo or balanced gradient echo/balanced steady-state free precession readouts, and single-shot fast-spin echo (FSE) are used [4], spin echo schemes can be preferable at higher field strength by limiting distortion. For multi-parametric examinations, it is advantageous to match the readout across the acquisitions in a multi-parametric protocol. This is especially relevant when combining T1 mapping with ASL, where T1 maps in the same data space may be used for the perfusion quantification in ASL [26], important in renal disease, where T1 significantly changes with the degree of fibrosis/inflammation. For matched readouts, a SE-EPI scheme provides the advantage that it can be for T1, ASL and DWI mapping. Usually, a transversal or coronal (oblique) readout is used [4] to capture both kidneys in one field of view, with a coronal orientation limiting through plane motion.

### Respiratory compensation

Adequate compensation for respiratory motion is of relevance since misalignment between acquisitions can introduce substantial errors in the calculated maps. Different strategies exist. For short acquisitions, breath-holding may suffice. However, especially in multiparametric acquisitions, the use of multiple breath-holds might be too challenging for patients. Alternatively, acquisitions can be aligned with respiration (respiratory triggering or gating) or paced breathing used preferably with post hoc motion correction. When fast single-shot 2D readouts are used, free-breathing acquisitions can be considered, enhancing patient comfort and decreasing scan time, but post hoc motion correction (image registration) is then mandatory. Underlying respiratory motion can induce signal fluctuations in the kidneys due to field (both B0 and B1) inhomogeneities in the abdomen, introducing additional noise. Furthermore, it can induce through-plane motion making motion correction more difficult. For T1 mapping, the inversion of contrast between source images makes image registration especially challenging, so some form of respiratory compensation during acquisition is advisable.

### B_0_ and B_1_ mapping

Another aspect to consider when performing T1 and T2 mapping is the collection of separate B0 and B1 maps to improve interpretation of data or to correct underlying inhomogeneities. B0 maps, computed from the phase difference between dual-echo gradient echo images, allow the assessment of off-resonance effects on image quality. B1 mapping allows the quantification of the local RF transmit (B1 +) field. In the abdomen, variations in image intensity can be notable due to B0 and B1 inhomogeneity, which can lead to significant differences between left and right kidneys. Non-ideal flip angles (in inversion pulse and readouts) can be included as a fitting parameter in the T1 fit or a separately acquired B1 map can be used in the fitting of the T1 data (as is required for the VFA method). Several B1 mapping methods have been developed, including the dual-TR or actual-flip angle (AFI) method [27], saturated double-angle method (SDAM) [28], dual refocusing echo acquisition mode (DREAM) [29], phase-sensitive method [30], Bloch–Siegert method [31] and use of a preconditioning RF pulse with turboFLASH readout [32]. Despite this, the commercial availability of B1 mapping schemes is limited, and there is no commonality in the natively availability of B1 mapping schemes across vendors, as highlighted in a recent paper to establish inter-vendor reproducibility of T1 relaxation times for brain imaging [33].

### Data analysis and reporting

Several data analysis and reporting steps are of relevance in renal T1 and T2 mapping.

#### Image registration

Prior to segmentation and ROI selection, it may be important to perform image registration across different TI times/VFA (T1 mapping) or echo times/preparation times (T2 mapping) to account for misalignment of slices due to abdominal motion. Motion correction can be performed using an affine registration or a deformable registration for severely motion affected slices.

#### Outlier detection and rejection

Outlier detection and rejection is crucial to avoid anomalous contributions from acquisition artifacts or motion-induced artifacts (seen as signal intensity errors across imaging slices) during data analysis. Outliers must be excluded from the dataset prior to ROI selection for correct estimation of T1/T2 values. Image registration techniques may help reduce outliers. In case of outliers due to imaging artifacts, care must be taken by excluding such slices from the analysis or data reporting.

#### Quantification

For each T1 mapping scheme, a different curve fitting function is used to obtain a T1 value. The Levenberg–Marquardt algorithm is the standard way to solve this nonlinear curve fitting problem. It should be noted that different estimation biases result depending on the fitting model such as number of parameters in the fit. T2 mapping sequences can be quantified by fitting a mono-exponential decay to the data. An overview of the T1 and T2 fitting functions and fitted parameters is presented in Table 1. Thermal and physiological noise, for example motion varies across subjects, will alter the model fitting, so it is important to determine the quality of the data. Robust estimation can be used to fit T1/T2 and estimate its standard deviation. This uses iterative re-weighting to improve the fit in the presence of outliers, at each iteration, the weighting of outliers is reduced based on the value of their residuals.

**Table 1:** Overview of functions used for quantification of T1 and T2 relaxation times

T1 mapping	
[48]	Classical inversion recovery (IR) T1 mapping Fitting of the classical inversion recovery(IR) mapping scheme for *M*_0_ and *T*_1_: $S_{k} = M_{0} \left( {1 - 2e^{{ - \frac{{TI_{k} }}{{T_{1} }}}} } \right)$ (1a) Assuming an ideal (100%) inversion
[13, 48]	Look Locker T1 mapping and variants such as Modified Look Locker T1 mapping (MOLLI) Data fit to a three-parameter nonlinear curve for *A*, *B* and $T_{1}^{*}$ $S_{k} = A - Be^{{ - \frac{{TI_{k} }}{{T_{1}^{*} }}}}$ (1b) with ‘true *T*_*1*_’ computed from: $T_{1} = T_{1}^{*} \left( {\frac{B}{A} - 1} \right)$
[14]	Variable Flip Angle T1 mapping Using multiple flip-angles, the *T*_*1*_ can be estimated by fitting for *M*_0_ and *T*_1_: $S_{k} = M_{0} \sin \alpha_{k} \frac{{\left( {1 - e^{{ - \frac{TR}{{T_{1} }}}} } \right)}}{{1 - \cos \alpha_{k} e^{{\left( { - \frac{TR}{{T_{1} }}} \right)}} }}$ (1c) By collecting the signal at different flip angles, T_1_ can be determined by first transforming Eq. (1c) into the linear form $\frac{{S_{k} }}{{\sin \alpha_{k} }} = E_{1} \frac{{S_{k} }}{{\tan \alpha_{k} }} + M_{0} \left( {1 - E_{1} } \right)$ where $E_{1} = e^{{ - \frac{TR}{{T_{1} }}}}$, and extracting T_1_ from the slope $m = E_{1}$ as $T_{1} = - \frac{TR}{{{ \ln }\left( m \right)}}$
T2 mapping	
[49]	Multi-echo spin echo sequence or T_2_ preparation modules The signal is defined using a mono-exponential decay fitting for *M*_0_ and *T*_2_ $S_{k} = M_{0} e^{{\left( { - \frac{{TE_{k} }}{{T_{2} }}} \right)}}$ (2)

*α* flip angle; *α*_k_, flip angle at *k*th pulse; *M*_0_, equilibrium magnetization; *M*_k_, magnetization at *k*th sampling pulse; *T*_1_, fitted pixel-by-pixel *T*_1_ values; *T*_1_*, apparent *T*_1_ (or modified *T*_1_ in the LL experiment); *T*_2_, fitted pixel-by-pixel *T*_2_ values; *T*_2_*, ‘observed’ *T*_2_ reflecting both true *T*_2_ as field inhomogeneities; *TE*_k_, multiple echo times/preparation times at *k*th TE scan time; *T*_D_, delay between flip angle and readout; TI, inversion recovery time; *TI*_k_, inversion recovery time at *k*th IR scan time; *S*_k_, the signal value at *k*th pulse

#### Reporting

The classical method of choice for reporting T1 and T2 mapping in the kidney involves manual ROI selection in the renal cortex and medulla on single/multiple slices, and/or different regions of the kidney (upper pole, interpolar and lower pole), with the combination of these yielding a single T1 or T2 value each for the cortex and the medulla, respectively. The main challenge of this method includes difficulty in drawing ROIs of an appropriate size and location avoiding partial volume effects when placed close to tissue interfaces, such as renal sinus fat and perirenal fat. Additionally, in the case of advanced renal disease such as chronic kidney disease (CKD), the cortico-medullary difference may become less apparent, due to alterations in the cortical and medullary T1 values, resulting in a decrease in cortico-medullary differentiation (CMD) [4]. Unclear boundaries due to reduced CMD may further introduce intra- and inter-rater bias when selecting ROIs. Alternative methods have been proposed in the literature for extracting the cortex and medulla using semi-automated or automated segmentation to reduce measurement variability and time. Semi-automated methods include histogram analysis [34] of the T1 map of the kidney, whereby the renal cortex and medulla is segmented by creating a histogram of T1 values across the kidney from which the two peaks can be used to separate cortex from medulla. Automated segmentation of the kidneys and its compartments (cortex, medulla, renal pelvis) based on registered T1- and T2-weighted images has been proposed by Will et al. [35], and machine learning methods are now being explored. However, such a technique will likely require co-registration of the T1- and T2-weighted images to either the T1/T2 mapping data.

## Materials and methods

### Description of survey process

The consensus project consisted of an approximation of a two-step modified Delphi method [36], which is a recommended approach to determine a reliable consensus in practice guidelines on health-care-related issues. This is outlined in more detail in the covering paper [5]. The Delphi method is an iterative process using repeated survey rounds to define consensus on proposed items and effective for determining expert group consensus on topics where there is little or no definitive evidence and where opinion is important [37]. Members of the PARENCHIMA Working Group 1.2 (Renal T1 and T2 mapping) and experts on renal MRI biomarkers based on recent literature were invited to participate in the Delphi panel. The survey process was conducted as described below.

#### Comparison of scan protocols from the literature and PARENCHIMA Working Group 1.2

A systematic literature search string for ‘renal T1 mapping’ and ‘renal T2 mapping’ was previously performed by the COST action PARENCHIMA and has been published elsewhere [4]. This systematic review aimed to provide an overview on potential clinical applications of the measurement of the independent quantitative magnetic resonance relaxation times T1 and T2 at both 1.5T and 3T. Information on scan protocols of published renal T1 and T2 mapping studies in the literature were used to identify key differences (e.g. field strength and sequences) between scan protocols that might limit pooling of data and future multicenter studies as a preparation for our electronic survey. In addition, members of the PARENCHIMA Working Group 1.2 (Renal T1 and T2 mapping) were asked to share detailed technical specifications of their local research T1 and T2 mapping protocols that were used in previous studies and/or unpublished work. In total, four T1 mapping protocols (Aarhus, Leiden, Leeds, Utrecht) and two T2 mapping protocols (Aarhus, Utrecht) were collected. Obtained T1 and T2 mapping protocols were tabulated to identify key differences and similarities between different research groups, different vendors, and different models of MR scanners and software versions. Results of the comparison of these T1 and T2 mapping scan protocols by the PARENCHIMA Working Group 1.2 members and the results of the systematic review on T1 and T2 mapping, served as a basis for the development of our electronic surveys.

#### Consensus formation

The Delphi method consisted of online surveys covering (a) hardware options and positioning, (b) in-plane spatial encoding, (c) spatial parameters, (d) RF and contrast, (e) customization and image analysis. Results of the first electronic survey round were discussed face-to-face in Aarhus, Denmark on March 18–19, 2019. Based on these discussions, follow-up survey questions were constructed for the second round, as well as to include questions on T2 mapping. In the follow-up electronic survey, Delphi panelists presented consensus statements based on the results of earlier versions of the electronic survey, which could be commented on by the panelists. Consensus was pre-defined as at least 75% consensus on the proposed question by the Delphi panel (excluding panelists who reported to have insufficient experience to make a recommendation with regard to the proposed question or statement). Survey questions in which over 40% of the Delphi panel noted to have insufficient experience to make a recommendation were excluded. Items that achieved consensus are discussed in the Results sections in the following order: patient preparation, hardware considerations, T1 mapping scheme, T2 mapping scheme, readout strategy, quantification, data analysis and reporting T1 and T2 values. An overview of the items asked in survey 1 and 2 are available online, as electronic supplementary material.

### Results and final recommendations

In total, 18 experts participated in the Delphi panel of which 9 responded in the first round and 17 in the second round, which meets the considered adequate number of experts for content validation [38]. Fourteen experts of the Delphi panel have a background in physics, three in clinical radiology, and one in nephrology. The first survey consisted of 56 items on renal T1 mapping of which four reached the pre-defined consensus threshold of 75% or higher. The second survey was expanded to include both renal T1 and T2 mapping, and consisted of 54 items of which 32 reached consensus. In the second round, five survey questions were excluded due to high number of experts reporting insufficient experience to make a recommendation. These five questions comprised survey statements on minimization of off-resonance, B1 maps, realignment and transformation of data acquired using breath hold scans, and whether outlier detection and rejection should be used. Nine experts noted that they collect both T1 mapping and ASL data routinely in their scan protocols. An overview of the items that reached consensus is provided in Table 2, and the recommendations arising from this process are discussed in the subsections below, indicated as “R” followed by a number (with an additional letter after this number if a recommendation belongs to the same category). In Table 3, a summary is provided of the most important recommendations.

**Table 2:** T1 and T2 mapping consensus based recommendations

No.	Consensus based recommendation	Consensus *n* (%)	Excluded *n* (%)
*Patient preparation*			
1	Subjects should be scanned in a normal hydration status when clinically appropriate	13 (87)	2 (12)
*Hardware*			
2a	T1 and T2 mapping can be performed at both 1.5T and 3T	T1 mapping: 6 (67) T2 mapping: 16 (94)	T1 mapping: 0 T2 mapping: 0
2b	A body coil transmitter and multi-channel receiver coil are hardware requirements for both T1 and T2 mapping	T1 mapping: 8 (100) T2 mapping: 16 (94)	T1 mapping: 1 (11) T2 mapping: 0
*Acquisition-general*			
3a	A look-locker variant is recommended as the T1 mapping scheme	16 (94)	0
3b	A minimum in-plane resolution of 3 mm is recommended for both Classic IR, MOLLI variant, and T2 mapping	Classic IR: 12 (92) MOLLI: 15 (100) T2 mapping: 15 (100)	Classic IR: 4 (24) MOLLI: 2 (12) T2 mapping: 2 (12)
3c	A parallel imaging factor of 2 is recommended for both Classic IR and MOLLI variant	Classic IR: 11 (85) MOLLI: 12 (80)	Classic IR: 4 (24) MOLLI: 3 (18)
3d	Collection of separate B0 and B1 maps when T1 or T2 maps are acquired is suggested	B0: 11 (92) B1: 12 (79)	B0: 5 (29) B1: 3 (18)
3f	A coronal or coronal oblique orientation are recommended for obtaining T1 and T2 maps of both kidneys during the same acquisition	5 (83)	3 (33)
*Acquisition-Classic IR*			
4a	Classic IR collected using an EPI readout with a minimum of 5 slices of 5 mm slice thickness are suggested scan parameters	5 (83)	3 (33)
4b	Considering renal T1 relaxation times, a minimum of 10 inversion times is suggested	11 (85)	4 (24)
4c	Classic IR data collected using respiratory triggering or paced breathing is suggested	14 (93)	2 (12)
4d	Classic IR data collected with right-left foldover is suggested	10 (83)	5 (29)
*Acquisition-MOLLI variant*			
5a	A shortened MOLLI scheme with a bFFE readout with 35° flip angle with a minimum slice thickness of 5 mm are suggested scan parameters	5 (83)	3 (33)
5b	A 5(3)3 MOLLI scheme is an acceptable sequence for renal T1 mapping	13 (100)	4 (24)
5c	MOLLI data should be collected with fixed spacing, i.e. ECG gating should not be used	11 (85)	4 (24)
5d	A fixed spacing of 1 s between RF pulses is suggested	13 (100)	4 (24)
5e	A minimum of one slice is sufficient for renal T1 mapping using MOLLI variant	11 (85)	4 (24)
5f	For clinical populations, collecting each slice in a single breath hold (BH) is suggested, a BH of less than 15 s is recommended	14 (93)	2 (12)
*Acquisition-T2 mapping*			
6a	A minimum of 5 echo times is suggested for data collection	13 (100)	4 (24)
6b	The recommended maximum echo time/T2 preparation time is at least the T2 relaxation time of the kidney (e.g. 120 ms at 3T)	14 (100)	3 (18)
*T1 quantification*			
7a	An inversion factor correction is not required in T1 quantification	10 (83)	5 (29)
7b	A B1 map can be of help to confirm good field inhomogeneity	11 (85)	4 (24)
7c	MOLLI T1 is quantified using a 3-parameter curve fit (*y* = *A* − B*exp(− *T*I/*T*1*) and correction (*T*1 = *T*1*(*B*/*A* − 1)) to yield T1	13 (100)	4 (24)
*Analysis of T1 and T2 values*			
8a	A manual ROI selection of the medulla and cortex is an acceptable analysis method	14 (88)	1 (6)
8b	When collecting multiple slices, combining all ROIs across all slices is suggested	12 (79)	3 (18)
8c	Automated ROI is preferred over manual ROIs	12 (79)	3 (18)
*Reporting of T1 and T2 mapping*			
9a	T1 and T2 values should be reported for cortex and medulla separately when possible and preferably contain either mean (standard deviation) or median (interquartile range)	Mean: 12 (79) Median: 14 (93)	Mean: 3 (18) Median: 3 (18)
9b	Reporting of the T1 cortex medulla difference (T1 medulla—T1 cortex) is suggested	15 (100)	2 (12)
9c	Reporting of the corticomedullary ratio (T1 cortex/T1 medulla) is suggested	13 (100)	4 (24)
9d	Reporting of number of cases without visible corticomedullary differentiation with regard to corresponding T1 and T2 values is recommended	15 (100)	2 (12)

**Table 3:** Final consensus recommendations on renal T1 and T2 mapping for patient preparation, acquisition, analysis and reporting

	T1 mapping	T2 mapping
Preparation	Normal hydration	Normal hydration
Field strength and hardware	1.5T or 3T, body coil transmitter and multi-channel receiver coil	1.5T or 3T, body coil transmitter and multi-channel receiver coil
Consensus sequence	MOLLI	MESE, GRASE, T2 prep^†^
Orientation	Coronal or coronal oblique	Coronal or coronal oblique
Acquisition MOLLI	≥ 1 slice, 3 mm in-plane resolution, slice thickness ≥ 5 mm, FA 35°, parallel imaging factor 2, 1 s fixed spacing, breath hold < 15 s	
Acquisition Classic IR	EPI readout, ≥ 5 slices, ≥ 10 inversion times, respiratory triggering or paced breathing, right-left foldover, parallel imaging factor 2	
Acquisition T2 mapping		≥ 5 echo times, max. TE/T2 prep time of 120 ms at 3T
Image quality control	Collection of B0 and B1 maps	Collection of B0 and B1 maps
ROI	Automated > manual, cortex and medulla, combining all ROIs across all slices	Automated > manual, cortex and medulla, combining all ROIs across all slices
Fitting	3-parameter curve fit (*y* = *A*−B*exp(− *TI*/*T*1*) and correction (*T*1 = *T*1*(*B*/*A*−1))	
Reporting	Cortex and medulla, T1 medulla—T1 cortex, T1 cortex/T1 medulla, number of cases without visible corticomedullary differentiation	Cortex and medulla, number of cases without visible corticomedullary differentiation
Reported metric statistics	Mean, median, standard deviation, interquartile range	Mean, median, standard deviation, interquartile range

^†^Consensus yet to be defined

#### Patient preparation

In the literature, different strategies for patient preparation have been described for renal T1 and T2 mapping, varying from no specific approach to several hours (2–6 h) of fasting. The expert panel recommended that subjects should be scanned in a normal hydration status when clinically appropriate **[R 1].** Little is known about the influence of hydration state on T1 or T2 values of the kidney; however, cardiac T1 mapping has shown that fluid overload significantly prolongs native T1 [39]. As fluid overload is also common in patients with renal disease, this can be an important confounder for the interpretation of native T1. No consensus was reached on whether diet needs to be controlled before scanning or whether subjects should follow a controlled or standardized salt intake. Factors for disagreement with the need for a diet control or standardized salt intake were practical limitations leading to difficulties in controlling diet or salt intake, particularly since data demonstrating a significant influence of diet on renal T1 or T2 is lacking.

#### Hardware considerations

Validation studies on renal T1/T2 mapping have been performed both at 1.5 and 3T, with recent multi-parametric studies being performed more frequently at 3T [4]. T1 and T2 mapping are acceptable be performed at both 1.5T and 3T **[R 2a]**. Higher field strength provides increased signal-to-noise ratio and greater dynamic range of T1 values, but conversely, there are greater field inhomogeneities and a shortened T2 dynamic range. The system-integrated body coil should be used for RF transmission and multichannel receivers are recommended when performing T1 and T2 mapping of the kidney **[R 2b]**, as implemented in cardiac and liver imaging.

#### T1 mapping scheme

For T1 mapping, a consensus was reached to recommend a Look-Locker variant (for example MOLLI) **[R 3a]**. This decision was reached as this is currently the only scheme that is widely available across MR vendors, with a 5(3)3 MOLLI scheme being an acceptable scheme **[R 5b]**. When a MOLLI scheme is chosen, a fixed spacing of 1 s is recommended as opposed to ECG triggering **[R 5c-d]**, as ECG triggering is not applicable to the kidney in contrast to cardiac imaging. Despite the MOLLI scheme being only a single slice method, this was agreed to be sufficient [**R 5e**] and should be collected in a breath hold of less than 15 s to be useful in patients that might be compromised in their ability to hold their breath for longer durations [**R 5f**]. 10 of the 17 experts (59%, no consensus) also recommended a classic inversion recovery based scheme comprising at least 10 different TIs **[R 4b]**. A VFA method is not recommended, only 20% of the panel felt this scheme is suitable for native T1 mapping.

The ultimate choice of T1 mapping scheme depends on the goal of the study. At the current time, MOLLI will provide an appropriate choice for a large multicenter study comprising a multiparametric protocol, since it is widely available and fast. However, for a single-center study aimed to detect subtle changes in tissue microstructure, one might choose the classical inversion recovery sequence (with or without slice cycling) which has been shown to be more precise over a wide range of T1 values [34].

#### T2 mapping scheme

For T2 mapping, no consensus was reached on a preferential scheme to use. MESE- or GRASE-based schemes are the preferred choice for large-scale studies due to being widely available but are slow. However, a *T2 prep* scheme, though not widely available, yields highly reproducible T2 measurements independent of scanner type and manufacturer, as shown for myocardial T2 mapping [40] and has the advantage that the same readout can be shared over multiple sequences (e.g. T1, T2 and ASL) within a multiparametric protocol [41].

A consensus was reached that at least five T2 weightings should be acquired for accurate T2 estimation **[R 6a]** and that the maximum echo time should be at least equal to the T2 of the kidney (e.g. approximately 120 ms at 3T) **[R 6b]**.

#### Readout strategy

In general, for a multiparametric scan protocol, it might be necessary, or at least convenient for data analysis and interrogation, to use the same readout for all acquisitions. In particular for T1 mapping and ASL, we recommend using the same readout, since the T1 maps can be used in the perfusion quantification [26].

For MOLLI, a single-shot balanced gradient echo/balanced steady-state free precession (bSSFP) readout with a flip angle of 35° is recommended **[R 5a]**, as this flip angle results in the highest signal-to-noise ratio. For classical inversion recovery, an EPI readout is recommended **[R 4a]**. No consensus was reached for T2 mapping.

Regarding spatial resolution, a minimal in-plane resolution of 3 mm is recommended **[R 3b]** to assess differences between cortex and medulla while maintaining signal-to-noise ratio. For T1 mapping, a maximum slice thickness of 5 mm is recommended **[R 4a, 5a],** but for T2 mapping, no consensus was reached. Regarding field of view and matrix size, no consensus was reached. For classical inversion recovery with an EPI readout, left–right phase encoding direction, as is typical for abdominal imaging, is recommended. A parallel imaging factor of 2 is recommended for T1 mapping readout schemes to yield high SNR, artifact-free maps.

A coronal or axial plane can be used to image both kidneys in the same field-of-view during one acquisition. However, a coronal or coronal oblique orientation (parallel to the long axis of the kidneys) is preferred **[R 3f]** as this orientation provides information about the distribution of T1 or T2 values in different anatomical areas of the kidney; upper pole, interpolar region, and lower pole. Furthermore, in this orientation, respiratory motion is in-plane and through plane motion is limited, enabling effective respiratory correction through registration. In contrast, for an axial acquisition, a given slice may be located at different levels in the kidney due to respiratory motion in free breathing acquisitions or inconsistent breath holds between multislice images.

#### Quantification

With regard to T1 quantification, a consensus was reached that inversion factor correction is not required **[R 7a]**. A B1 map can be beneficial for confirming good field homogeneity **[R 7b]**, though the need for a B1 map to correct for the readout flip angle (e.g. to ensure the exact flip angle is used in MOLLI scheme) is still debatable. Limitations of additional B0/B1 mapping increasing the technical complexity to the scan protocol were raised. For the MOLLI scheme, a consensus was reached that T1 values should be quantified using a three-parameter curve fit (Eq. [1d]) **[R 7c]**.

#### Data analysis and reporting T1 and T2 values

For image analysis, the expert panel considered manual ROI selection of the medulla and cortex to be an acceptable analysis method at this moment **[R 8a]**. However, the expert panel considered automated ROIs to be preferred over manual ROIs **[R 8c]**. For protocols that acquire multiple slices of the kidney in the same orientation, it is recommended to combine all ROIs across all slices **[R 8b]** to reach a more balanced estimate of the ROI measurement. No consensus was reached on whether single or multiple ROIs in the cortex or medulla be used, or on the need for taking ROI size into account when using multiple ROIs.

Several recommendations were made by the expert panel with regard to the reporting of T1 and T2 mapping results. In subjects with visible corticomedullary differentiation, relaxation times should be provided for cortex and medulla separately **[R 9a]**. In addition, T1 and T2 values should be reported as either mean with corresponding standard deviation or median with interquartile range (depending on the distribution of the data). Suggested measures to reflect corticomedullary differentiation are both the T1 cortex medulla difference (T1 medulla—T1 cortex) **[R 9b]** and the corticomedullary ratio (T1 cortex/T1 medulla) **[R 9c]**. It is recommended to report the number of cases with no visible corticomedullary differentiation, as this limits the determination of separate relaxation times for renal cortex and medulla **[R 9d]**.

## Discussion

### Issues not reaching consensus

No T2 mapping sequence (MESE, GRASE, T2 preparation module) reached consensus. In addition, no consensus was reached with regard to a minimum matrix size for renal T1 and T2 mapping schemes. Noting that for an EPI acquisition, the minimum achievable echo time is dependent on the matrix size and acceleration factor used, it is suggested that a minimum field of view of 320 mm × 320 mm be considered to ensure a reasonable echo time for classic IR T1 mapping. With regard to adopting methods to minimize off-resonance effects to avoid banding artifacts in MOLLI variants of T1 mapping, 53% of the panel had insufficient experience to make a recommendation so no consensus was reached. To provide some guidelines, B0 shimming and centre frequency can be adjusted to minimize off-resonance. This is especially important at higher field strengths where off-resonance effects can result in regional variations in apparent T1 [12]. If available, B1 shimming also improves both T1 and T2 estimation.

Although consensus was reached that manual ROI analysis of the renal cortex and medulla is acceptable, no specific strategy was decided upon from the following strategies: one large ROI parallel to the outer edge of the cortex; at least three ROIs of > 0.1 cm^2^ in representative areas of both cortex and medulla; ROIs in upper pole, interpolar, lower pole region of both kidneys; and one ROI including, respectively, the cortex or medulla as a whole. In addition, it was highlighted that studies on the reproducibility of manual ROI measurement of renal T1 and T2 mapping are needed. For automated ROI analysis, no specific strategy resulted from the questionnaire. Automated ROI analysis strategies mentioned by the expert panel included a visual distribution approach (e.g. k-means clustering), and histogram analysis to differentiate between cortex and medulla. Furthermore, it was highlighted that heterogeneity in the distribution of T1/T2 values across the kidney may be useful for assessing the presence and progression of CKD.

### Limitations and remaining challenges for future research

The panel of experts that participated in this consensus formation process was of limited size (*n* = 18), which can be considered a shortcoming of this work. However, it included scientists from groups that have all developed or applied renal T1 mapping applications. The proportion of technically oriented panel members was high, justified by the current state of development of the technique. Other limitations include differences in the level of detail in the provided recommendations as these are inherent to the maturity of the research field. As such, provided recommendation on data analysis and reporting include semi-automated approaches, and the influence of fitting routines, defining of outliers, handling of missing slice data, and the associated penitential bias for estimated T1 and T2 have not been addressed. Several knowledge gaps are highlighted based on the results of this survey. More research is needed on possible factors influencing renal T1 and T2 measurements such as hydration state, fasting state, salt intake or medication use, with hydration state being of great interest as volume regulation can be affected in renal patient populations. Despite reaching consensus on the 5(3)3 MOLLI scheme for renal T1 mapping, this scheme has been optimized for cardiac T1 mapping and its use is in part driven by its availability across all major MR vendors, rather than its optimization for measurement of renal T1 values, leaving room for further improvement. Furthermore, the use of a 5(3)3 MOLLI scheme can be limited with respect to spatial resolution, since each of the 5,3,3 single-shot images must occur within a 1 s interval. High spatial resolution MOLLI data can be achieved through the use of segmented multi-shot data acquisitions, assuming each breath-hold is consistent. Likewise, it should be noted that a classic IR with single-shot EPI acquisition is also limited in achievable spatial resolution due to the increased echo time at higher spatial resolution.

Although T1 values of renal cortex, medulla, and corticomedullary ratio have proven to be highly reproducible for both classic IR and MOLLI 5(3)3 schemes [9, 42, 43], no studies thus far have evaluated intra- and inter-observer reproducibility of manual and (semi)automated analysis strategies for the assessment of T1 values in the kidney. In addition, reproducibility studies on renal T2 mapping are lacking. Moreover, the survey responses underline the need for dedicated renal post-processing software to facilitate automated image analysis of T1 and T2 values in cortex and medulla and provide quantitative error estimates for reliability assessment, key for use in clinical decision making [44]. Besides uniformity in scan protocols, high-quality healthy volunteer reference data are needed to define a reference range, as has been recently published for cardiac T1 mapping [45], and which requires sufficiently large cohorts to reflect normal variations. Since T1 and T2 mapping sequences have specific precision and measurement errors, data collected in patient populations should be compared with normal reference values obtained using the same mapping scheme (pulse sequence parameters and field strength) [46]. Multicentre studies require verification on whether the scanner configurations are identical [47] and phantom validation is essential component of intra- and inter-vendor validation prior to performing a multicentre study.

## Conclusion

Technical recommendations were constructed to incorporate the opinions and advice of a multidisciplinary group on renal T1 and T2 mapping. These highlight the current lack of consensus in both renal T1 and T2 mapping, to some extent surprising considering the long history of relaxometry in MRI, highlighting key knowledge gaps that require further work. Given the dynamic nature of physiological imaging methods in terms of data acquisition and analysis, we expect and encourage detailed studies to systematically compare renal T1 and T2 mapping methods, and validate methods against reference standards for inter-site studies, and harmonize approaches across vendors. This paper should be regarded as a first step in a long-term evidence-based iterative process towards ever increasing harmonization of scan protocols across sites. These outcomes should inform periodic updates of these recommendations on renal T1 and T2 mapping. The panel will stay in existence and recommendations will be revisited and updated as and when new evidence becomes available.

## Electronic supplementary material

Below is the link to the electronic supplementary material.

Supplementary material 1 (DOCX 30 kb)

Supplementary material 2 (DOCX 22 kb)
