# Supplementary material for: Consensus-based technical recommendations for clinical translation of renal T1 and T2 mapping MRI
Source: MAGMA. 2019 Nov 22;33(1):163–76. doi: 10.1007/s10334-019-00797-5 (PMC7021750; doi:10.1007/s10334-019-00797-5)
Supplement: Supplementary file 1 — Supplementary material 1 (DOCX 30 kb) [file 10334_2019_797_MOESM1_ESM.docx]

**Online supplemental- survey 1**

| **Question** | **Answers** | | | | | | | |
| --- | --- | --- | --- | --- | --- | --- | --- | --- |
| **Patient preparation** |  |  |  |  | | |  | |
| 2.1 Fasting before scanning? | Yes  3 | No  3 | Don’t’ know  0 | No recommendation  2 | | | Other  1 | |
| 2.2 Restriction on liquids before scanning? | Yes  3 | No  1 | Don’t’ know  1 | No recommendation  1 | | | Other  3 | |
| 2.3 Any other patient preparation procedure? | Yes  0 | No  4 | Don’t’ know  0 | No recommendation  2 | | | Other  3 | |
| Other topics to be included on patient preparation? | Open answers | | | | | | | |
| **Hardware** |  |  |  |  | | | | |
| 3.1 Field strength? | 1.5T  0 | 3T  2 | Both  6 | Other  1 | | | | |
| 3.2 Transmitter coil? | Body  9 | | | No recommendation  1 | | | Other  0 | |
| 3.3 Receiver coil? | Body  8 | | | No recommendation  1 | | | Other  0 | |
| Other topics to be included on hardware? | Open answers | | | | | | | |
| **T1 mapping scheme** |  | |  |  |  | |  | |
| 4.1 T1 mapping scheme? | Classic IR  1 | | LL-variant  5 | VFA  1 | SASHA  1 | | No recommendation  1 | |
| **Classic IR** |  |  | |  | | |  | |
| 5.1 Inversion pulse type? | FOCI  1 | Hyperbolic secant  1 | | No recommendation  4 | | | Do not know  3 | |
| 5.2 Number of inversion times to use ? [choose a single option – state inversion times and field strength used in ‘other’] | 5  3 | 11  1 | 13  1 | No recommendation  2 | | | Do not know  1 | |
| 5.3 Breathing scheme? | BH  1 | RT  1 | FB  1 | No recommendation  3 | | | Do not know  1 | Other  2 |
| 5.4 Image readout type? | 2D-multisclice  2 | | 3D  2 | No recommendation  4 | | | Do not know  1 | |
| 5.5 Slice orientation? | Cor/Cor-oblique  5 | Sag  0 | Tra  0 | No recommendation  2 | | | Do not know  1 | Other  1 |
| 5.6 Readout sequence for 2D? | SE-EPI  2 | GRE-EPI  1 | bSSFP  0 | No recommendation  4 | | | Do not know  1 | |
| 5.7 Number of slices in 2D? | 2-4  1 | 4-6  2 | >10  1 | No recommendation  3 | | | Do not know  2 | |
| 5.8 Slice thickness for 2D? | 5mm  3 | 6mm  2 | 8mm  1 | No recommendation  3 | | | Do not know  2 | |
| 5.9 Slice gap in 2D multi-slice? | 0.5-1mm  1 | | No gap  2 | No recommendation  4 | | | Do not know  2 | |
| 5.10 Readout sequence for 3D? | 3D-RARE  1 | 3D-GRASE  1 | | No recommendation  5 | | | Do not know  3 | |
| 5.11 Use segmented 3D sequences? | Yes  1 | No  0 | Don’t’ know  3 | No recommendation  5 | | | | |
| 5.12 Number of slices in 3D? | 5-10  slices  0 | 10-15  0 | 15-20  slices  2 | No recommendation  4 | | | Don’t’ know  3 | |
| 5.13 Slice thickness for 3D? | 3mm  1 | 4mm  1 | Other  0 | No recommendation  4 | | | Don’t’ know  3 | |
| 5.14 Required FOV of image? | 244x244  1 | 300x300  1 | 400x  400  2 | No recommendation  4 | | | Don’t’ know  1 | |
| 5.15 In-plane resolution? | 2mm  1 | 3mm  3 | Other  0 | No recommendation  4 | | | Don’t’ know  1 | |
| 5.16 Partial Fourier phase-encoding? | None  1 | 80-90%  1 | Other  0 | No recommendation  6 | | | Don’t’ know  1 | |
| 5.17 Partial Fourier slice (partition)-encoding in 3D? | 60-70%  0 | 70-80%  0 | 80-90%  0 | No recommendation  6 | | | Don’t’ know  3 | |
| 5.18 Use parallel imaging? [choose a single option] | Yes  3 | No  0 | Don’t’ know  1 | No recommendation  5 | | | | |
| 5.19 Parallel imaging acceleration factor? | 1.5  1 | 2  2 | 3  0 | No recommendation  5 | | | Don’t’ know  1 | |
| 5.20 TR (between inversion pulses)? | 4-5s  1 | 6-7s  3 | 8-9s  0 | No recommendation  4 | | | Don’t’ know  1 | |
| Other topics relevant to the use of a Classic Inversion Recovery Scheme? | Open answers | | | | | | | |
| **MOLLI variant** |  |  |  |  | | |  | |
| 6.1 Inversion pulse type? | FOCI  0 | Hyperbolic secant  3 | | No recommendation  2 | | | Do not know  4 | |
| 6.2 Look Locker variant to use | 5(3)3  3 | 5(1)1(1)1  1 | 16-8-4  1 | No recommendation  2 | | | Do not know  3 | |
| 6.3 Do you ECG trigger or use physiological simulation? | ECG  2 | Simulaton 60 bpm  3 | Other  1 | No recommendation  1 | | | Do not know  3 | |
| 6.4 What MOLLI flip angle should be used? | 12°  1 | 35°  1 | Other  0 | No recommendation  2 | | | Do not know  3 | |
| 6.5 Slice orientation? | Cor/Cor-oblique  7 | Sag  0 | Tra  0 | No recommendation  1 | | | Do not know  1 | |
| 6.6 Required FOV of image? | 300x300  1 | 400x400  4 | Other  0 | No recommendation  3 | | | Do not know  1 | |
| 6.7 In-plane resolution? | 1mm  1 | 2mm  3 | 3mm  2 | No recommendation  2 | | | Do not know  1 | |
| 6.8 Breathing scheme? | BH  4 | FB  0 | Other  0 | No recommendation  2 | | | Do not know  3 | |
| 6.9 Is Minimization of off-resonance effects performed? | Main field shim and center frequency  0 | | | No recommendation  3 | | | Do not know  5 | |
| 6.10 Is the MOLLI scheme repeated for a number of slices? | 1  1 | 2  1 | 3-5  2 | No recommendation  3 | | | Do not know  4 | |
| **VFA variant** |  |  |  |  | | |  | |
| 7.1 How many flip angles do you use? | 2  2 | 5  1 | Other  0 | No recommendation  3 | | | Do not know  3 | |
| 7.2 Slice orientation? | Cor/Cor-oblique  4 | Sag  0 | Tra  0 | No recommendation  2 | | | Do not know  3 | |
| 7.3 Required FOV of image? | 380x380  1 | 400x400  1 |  | No recommendation  4 | | | Do not know  3 | |
| 7.4 In-plane resolution? | 1mm  1 | 2mm  1 | 3mm  1 | No recommendation  2 | | | Do not know  4 | |
| 7.5 Breathing scheme? | BH  3 | FB  0 | Other  0 | No recommendation  2 | | | Do not know  4 | |
| Other topics relevant to the use of a VFA variant? | Open answers | | | | | | | |
| **B0 & B1 map sequence** |  |  |  |  | | |  | |
| 8.1 Do you collect a separate B0 and B1 map sequence? | Yes  3 | No  4 | Other  1 | No recommendation  0 | | | Do not know  1 | |
| 8.2 Which B0 mapping scheme? | Dual-echo GRE  2 | 6-echo  Dixon  1 | | No recommendation  3 | | | Do not know  3 | |
| 8.2 Which B1 mapping scheme? | DREAM  1 | Dual TR-AFI  1 | Other  0 | No recommendation  4 | | | Do not know  3 | |
| 8.3 Slice orientation? | Cor/Cor-oblique  5 | Sag  0 | Tra  0 | No recommendation  1 | | | Do not know  3 | |
| 8.4 Breathing scheme? | BH  3 | FB  0 | Other  1 | No recommendation  2 | | | Do not know  3 | |
| 8.5 Motion compensation? | None  1  1 | Expiration respiratory triggering  2 | Navigators  2 | No recommendation  1 | | | Do not know  3 | |
| Other topics relevant to the use of a B1 map sequence? | Open answers | | | | | | | |
| **Data pre-processing** |  |  |  |  | |  |  | |
| 9.1 Motion correction? | None  3 | Rigid  1 | Affine  3 | Non-rigid  2 | | No recommendation  0 | Do not know  0 | |
| 9.2 Outlier detection and rejection? | Yes  3 | No  4 | No recommendation  1 | | | | Do not know  1 | |
| Other topics relevant to data preprocessing? | Open answers | | | | | | | |
| 10 Is your T1 mapping protocol also used for quantification of ASL | Yes  4 | No  5 |  |  | | |  | |
| Other topics relevant to collecting T1 mapping for quantification of ASL? | Open answers | | | | | | | |
| **Quantification of T1 values** |  |  |  |  | | |  | |
| 11.1 Do you use B1 mapping in T1 quantification? | Yes  1 | No  6 | Do not know  0 | No recommendation  1 | | | Other  1 | |
| 11.2 Do you use an inversion factor correction in your T1 quantification? | Yes  1 | No  5 | No recommendation  3 | | | | Do not know  0 | |
| 11.3 Which method do you use for MOLLI T1 quantification? [choose a single option]? | Original fit  4 | | Other  0 | No recommendation  2 | | | Do not know  3 | |
| Other topics relevant to quantification of T1 values? | Open answers | | | | | | | |
| **Reporting T1 values** |  |  |  |  | | |  | |
| 12.1 Region of interest selection method (cortex, and medulla)? | Manual  5 | TLCO  1 | k-means  1 | Other  1 | | | No recommendation  1 | |
| 12.2 Region of interest measurements? | Mean+SD  5 | Mode+FWHM  1 | Other  1 | No recommendation  1 | | | Do not know  1 | |
| 12.3 Measurements for corticomedullary differentiation ? | C-M ratio  3 | C-M difference  1 | 100x (C-M)/C  1 | No recommendation  2 | | | Do not know  2 | |
| 13.1 Please enter any comments on the questionnaire or suggestions (e.g. additional questions) | Open answers | | | | | | | |
