# Supplementary material for: Consensus-based technical recommendations for clinical translation of renal T1 and T2 mapping MRI
Source: MAGMA. 2019 Nov 22;33(1):163–76. doi: 10.1007/s10334-019-00797-5 (PMC7021750; doi:10.1007/s10334-019-00797-5)
Supplement: Supplementary file 2 — Supplementary material 2 (DOCX 22 kb) [file 10334_2019_797_MOESM2_ESM.docx]

**Online supplemental 2**

| **Question** | **I agree** | | **I disagree** | | **Insufficient experience to recommend** |  |
| --- | --- | --- | --- | --- | --- | --- |
| **2 Patient preparation**  2.1 Diet needs to be controlled before the scan. | 4 | | 6 | | 4 |  |
| 2.2 Subject should be scanned in a normal hydration status when clinically appropriate. | 13 | | 0 | | 2 |  |
| 2.3 Subjects are required to follow a controlled and standardized salt intake before the scan | 2 | | 8 | | 5 |  |
| **3 Hardware** |  | |  | |  |  |
| 3.1 Based on the previous questionnaire the following consensus statement was formulated: T1 mapping can be performed at both 1.5T and 3T. If there are any disagreements to this please specify below. | Open answer | | | | | |
| 3.2 Based on the previous questionnaire the following consensus statement was formulated: T1 mapping should be performed using a body coil transmitter and receiver with a minimum of 18 channels. If there are any disagreements to this please specify below. | Open answer | | | | | |
| **4 T1 mapping scheme** |  | | | | | |
| 4.1 T1 Mapping scheme – NOTE: you can recommend more than one scheme (In comments: please specify order of preference if you recommend more than one readout scheme) | Classic IR  10 | | Look-Locker variant  16 | | VFA  3 |  |
| *5 Classic Inversion Recovery scheme* |  | |  | |  |  |
| 5.1 classic IR scheme - Based on the previous questionnaire the following consensus statement was formulated: Classic IR scheme should be collected using an EPI readout with a minimum of 5 slices of 5 mm slice thickness in a coronal or coronal oblique orientation (preferred when combined with ASL). If there are any disagreements to this please specify below. | Open answer | | | | | |
| 5.2 classic IR scheme - A minimum of 10 inversion times should be used (please state inversion times you recommend in other). | 11 | | 1 | | 4 |  |
| 5.3 classic IR scheme - Data should be collected using respiratory triggering or using paced breathing. | 14 | | 1 | | 2 |  |
| 5.4 classic IR scheme - Data should be collected with foldover in: RL, FH, no recommendation, no experience | RL  10 | | 0 | | 5 |  |
| 5.5 classic IR scheme - Data should be collected with 3 mm in-plane resolution | 12 | | 1 | | 4 |  |
| 5.6 classic IR scheme - Noting that for a EPI acquisition the minimum achievable echo time is dependent on the matrix size and acceleration factor that can be used. Data should be collected with a minimum matrix of 320 x 320 matrix to ensure a reasonable echo time. | 7 | | 2 | | 5 |  |
| 5.7 classic IR scheme - A parallel imaging factor of 2 should be used. | 11 | | 1 | | 4 |  |
| 5.8 Please specify any additional information you wish to include about implementation of a classic IR scheme. | Open answer | | | | | |
| *6 MOLLI variant* |  | | | | | |
| 6.1 MOLLI variant - Based on the previous questionnaire the following consensus statement was formulated: A shortened MOLLI scheme with a bFFE readout with 35o flip angle should be collected using a coronal or coronal oblique (preferred when combined with ASL) slice of 5 mm slice thickness. If there are any disagreements to this please specify below. | Open answer | | | | | |
| 6.2 MOLLI variant - A 5(3)3 shMOLLI scheme is an acceptable for renal T1 mapping (if no, please state what scheme you recommend). | 13 | | 0 | | 4 |  |
| 6.3 MOLLI variant - shMOLLI data should be collected with fixed spacing, i.e. ECG gating should not be used. | 11 | | 2 | | 4 |  |
| 6.4 MOLLI variant - Since the precision of the T1 estimate can be altered by the spacing of the RF pulses (as stated in https://doi.org/10.1186/1532-429X-16-2), a fixed spacing of 1s between RF pulses should be used (if you recommend a different spacing please state this). | 11 | | 0 | | 5 |  |
| 6.5 MOLLI variant - The recommended in-plane resolution is 2-3 mm. | 15 | | 0 | | 2 |  |
| 6.5 MOLLI variant - A parallel imaging factor of 2 should be used. | 12 | | 2 | | 3 |  |
| 6.6 MOLLI variant - Data should be collected with a 400 x 400 matrix. | 8 | | 4 | | 5 |  |
| 6.7 MOLLI variant - A minimum of one slice is sufficient (if no, please include number of slices, slice thickness and gap in comments). | 10 | | 1 | | 3 |  |
| 6.8 MOLLI variant - Each slice should be collected in a single breath hold (BH) for clinical populations, a BH of less than 15 s is recommended. | 14 | | 1 | | 2 |  |
| 6.9 MOLLI variant - Minimization of off-resonance effects to avoid banding artefacts should be performed (if yes, please specify the method you use to minimize banding effects). | 7 | | 0 | | 9 |  |
| 6.9 MOLLI variant - Please specify any additional information you wish to include about the implementation of a Look Locker scheme variant (i.e. LL, MOLLI, shMOLLI). | Open answer | | | | | |
| 7.1 T1 Quantification - An inversion factor correction is not required in T1 quantification (e.g. for a classic inversion you assume 100 % inversion efficiency and fit to (1-2*exp(-TI/T1)). | 10 | | 2 | | 5 |  |
| 7.2 T1 Quantification - A B1 map should be acquired and used to confirm good field inhomogeneity. | 11 | | 2 | | 4 |  |
| 7.3 T1 Quantification - A B1 map should be used in T1 quantification to correct for the readout flip angle (e.g. to account for the exact flip angle used in shMOLLI scheme) (if yes, please add to the comments which T1 mapping scheme and which vendor you use) | 3 | | 3 | | 9 |  |
| 7.4 T1 Quantification - shMOLLI T1 is quantified using a 3-parameter curve fit and correction to yield T1 (i.e. original fit method (3-parameter exponential curve fitting (y=A-B*exp(-TI/T1*)) and correction T1=T1*(B/A-1))). | 13 | | 0 | | 4 |  |
| 7.4 Please specify any additional information you wish to include about T1 quantification. | Open answer | | | | | |
| Do you also collect T1 mapping data for ASL quantification? | Yes  9 | | No  8 | | 0 |  |
| 9.1 T2 mapping hardware - T2 mapping can be performed at any field strength, using a body coil transmitter and receiver with a minimum of 18 channels. | 16 | | 1 | | 0 |  |
| 9.2 T2 Mapping scheme – NOTE: you can recommend more than one scheme (please specify order of preference in you recommend more than one readout scheme or add your own recommendation) | MESE (fast)  8 | T2prep  6 | | MESE EPI  3 | 4 |  |
| 9.3 T2 Mapping acquisition – Recommended in-plane resolution is 2-3 mm in-plane resolution. | 15 | | 0 | | 2 |  |
| 9.4 T2 Mapping acquisition – Data should be collected with a 400 x 400 matrix. | 10 | | 5 | | 2 |  |
| 9.5 T2 Mapping acquisition – Data should be collected with a minimum of 5 echo times (please add minimum echo times you recommend in other) | 13 | | 0 | | 4 |  |
| 9.6 T2 Mapping acquisition – The maximum echo time/T2 preparation time should be at least the T2 relaxation time of the kidney (e.g. 120 ms at 3T https://doi.org/10.1093/ndt/gfy198) | 14 | | 0 | | 3 |  |
| 9.7 T2 Mapping acquisition – Please specify any additional information you wish to include | Open answer | | | | |  |
| 10.1 For breathhold scans, data should be realigned using a: | Rigid  6 | | Affine  4 | | 7 |  |
| 10.2 For free breathing/respiratory triggered/paced breathing a affine/deformable transformation should be used: | 9 | | 1 | | 7 |  |
| 10.3 Outlier detection and rejection should be used (if yes, please specify which outlier detection and rejection method you use). | 8 | | 1 | | 8 |  |
| 11.1 A separate B0 map should be collected in the protocols when collecting T1 or T2 maps. | 11 | | 1 | | 5 |  |
| 11.2 A separate B1 map should be collected in the protocols when collecting T1 or T2 maps. | 12 | | 2 | | 3 |  |
| 12.1 Manual ROI - A manual ROI selection of the medulla and cortex is an acceptable analysis method (if no, please specify which method you are using and why). | 14 | | 0 | | 1 |  |
| 12.2 Manual ROI - For a given kidney, you should assess multiple ROIs in the cortex (please state size of ROIs and number used and the way of distributing these in the kidneys in comments). | 6 | | 3 | | 1 |  |
| 12.3 Manual ROI - For a given kidney, you should assess multiple ROIs in the medulla (please state size of ROIs and numbers used and the way of distributing these in comments). | 7 | | 2 | | 2 |  |
| 12.4 Manual ROI - For a given kidney, you should assess a single ROI in the cortex (please state how you outline this ROI in comments). | 2 | | 8 | | 1 |  |
| 12.5 Manual ROI - For a given kidney, you should assess a single ROI in the medulla (please state how you outline this ROI in comments). | 1 | | 11 | | 1 |  |
| 12.6 Manual ROI - When collecting multiple slices, you should combine all ROIs across all slices. | 12 | | 2 | | 3 |  |
| 12.7 Manual ROI - When collecting multiple ROIs you should account for size of ROIs when calculating final result. | 8 | | 5 | | 4 |  |
| 12.8 Automated ROI - An automated ROI should be generated (if yes, please state how this is generated and how it is used in CKD patients when corticomedullary differentiation is reduced). | 8 | | 2 | | 4 |  |
| 12.9 Automated ROI - Automated ROI is preferred over manual ROIs. | 12 | | 2 | | 3 |  |
| 13.1 When computing resultant T1 or T2 values you should compute mean and standard deviation. | 12 | | 2 | | 3 |  |
| 13.2 When computing resultant T1 or T2 values you should compute median and IQR. | 13 | | 1 | | 3 |  |
| 13.5 The T1 corticomedullary differentiation should be computed. | 15 | | 0 | | 2 |  |
| 13.6 The corticomedullary ratio (T1 cortex / T1 medulla) is useful as a measure of corticomedullary differentiation. | 13 | | 0 | | 4 |  |
| 13.7 The corticomedullary difference (T1 medulla - T1 cortex) is useful as a measure of corticomedullary differentiation. | 13 | | 1 | | 3 |  |
| 13.8 You should report how you handle cases without visible corticomedullary differentiation (please add further details in comments) | 15 | | 0 | | 2 |  |
